# Correction: Dou et al. Performance Calibration of the Wavefront Sensor’s EMCCD Detector for the Cool Planets Imaging Coronagraph Aboard CSST. *J. Imaging* 2025, *11*, 203

**DOI:** 10.3390/jimaging11090303

**Published:** 2025-09-05

**Authors:** Jiangpei Dou, Bingli Niu, Gang Zhao, Xi Zhang, Gang Wang, Baoning Yuan, Di Wang, Xingguang Qian

**Affiliations:** 1 Nanjing Institute of Astronomical Optics & Technology, Chinese Academy of Sciences, Nanjing 210042, China; 2 CAS Key Laboratory of Astronomical Optics & Technology, Nanjing Institute of Astronomical Optics & Technology, Nanjing 210042, China; 3 University of Chinese Academy of Sciences, Beijing 100049, China

The authors would like to make the following corrections to the published paper [1]. The changes are as follows:

(1) In the Abstract and Section 1 Introduction, Paragraph 2, the full form of CSST has been updated to “Chinese Space Station Survey Telescope (CSST)”.
(2) In the Section 1 Introduction, Paragraph 2, the corrected elements should read as follows:
  “CPI-C addresses this issue by utilizing a multi-channel electron-multiplying charge-coupled device (EMCCD) in its wavefront sensor.”
(3) In the Section 2.1, the corrected elements should read as follows:
  “The detector consists of three core components: (a) an imaging assembly integrating the multi-channel EMCCD chip for optical signal acquisition,”
(4) In Figure 1 and Sections 2.2 and 4, the authors have replaced all model-specific references to the detector chip with the generic term “EMCCD”.

In the original publication, there was a mistake in Figure 2 and Table 1. This correction appears as follows.

(1) Figure 2
  A revised version of Figure 2 is provided with an updated caption. The reference [28] previously cited in conjunction with Figure 2 has been removed accordingly. The updated figure and caption are as follows:

**Figure 2:**
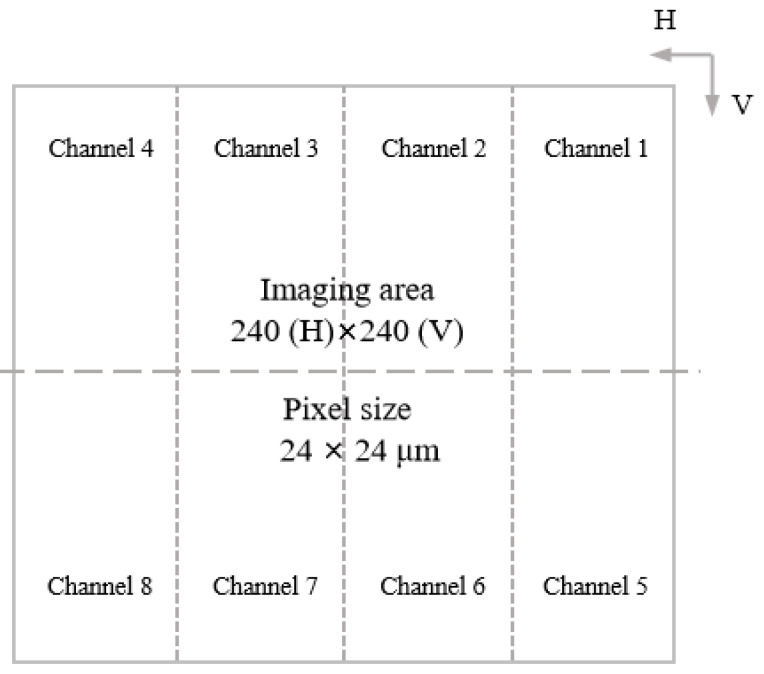
EMCCD focal plane schematic: Storage section with 8 parallel readout channels, each channel: 60 (H) × 120 (V) pixels, and output amplifiers shared pairwise (channels 1–2, 3–4, 5–6, 7–8).
(2) In Table 1, the authors wish to revise the CCD entry to “8-channel EMCCD”. Simultaneously, the information concerning camera weight in the last row of Table 1 should also be deleted, as this parameter is not substantially pertinent to the core theme of the article. The corrected elements should read as follows:

**Table 1:** Key design specifications of the detector.

Specifications	Value
CCD type	8-channel EMCCD
Image readout quantization number	14 bit
Exposure time	3 ms~5 s, adjustment interval 1 ms
EM Gain	Max value ≥ 100×

Regarding the Funding section, the corrected elements should read as follows:

**Funding:** This research was supported by the National Natural Science Foundation of China (grants nos. 11827804 and U2031210) and the China Manned Space Project (grants nos. CMS-CSST-2025-A18, CMS-CSST-2021-A11, CMS-CSST-2021-B04, and CMS-CSST-201906). Furthermore, this study is part of a project funded by the Natural Science Foundation of Jiangsu Province (grant no. BK20241708) and received support from the China Postdoctoral Science Foundation under grant number 2024M763371.

The authors state that the scientific conclusions are unaffected. This correction was approved by the Academic Editor. The original publication has also been updated.
